# Supplementary protection certificates and their impact on access to medicines in Europe: case studies of sofosbuvir, trastuzumab and imatinib

**DOI:** 10.1186/s40545-019-0198-6

**Published:** 2020-01-14

**Authors:** Yuanqiong Hu, Dimitri Eynikel, Pascale Boulet, Gaelle Krikorian

**Affiliations:** 1 MSF Access Campaign, Médecins Sans Frontières, Rue de Lausanne 78, P. O Box 1016, CH-1211 Geneva 1, Switzerland; 2 School of Law, School of Oriental and African Studies (SOAS), University of London, Russell Square, 10 Thornhaugh Street, Russell Square, London, WC1H OXG UK; 3 MSF Access Campaign, Médecins Sans Frontières, Rue de Lausanne 78, P. O Box 1016, CH-1211 Geneva 1, Switzerland; 4 Medicines Law & Policy, 105 route de Lossiège, 74130 Contamine sur Arve, France; 5 MSF Access Campaign, Médecins Sans Frontières, Rue de Lausanne 78, P. O Box 1016, CH-1211 Geneva 1, Switzerland

**Keywords:** Access to medicines, Supplementation protection certificate (SPC), Drug prices, Intellectual property, Generic competition, European Union

## Abstract

In recent years, there has been increasing pressure on public health systems in high-income countries due to high medicines prices, one of the underlying causes of which are the market monopolies granted to pharmaceutical undertakings. These monopolies have been facilitated by expanded forms of intellectual property protections, including the extension of the exclusivity period after the expiration of the patent term concerning medicinal products. In the European Union such an approach lies in the Supplementary Protection Certificate, a mechanism formally introduced under Regulation 1768/92/EEC (now: Regulation 469/2009/EC, amended). After more than 20 years of implementation since it was first introduced, the common justifications for SPCs are being challenged by recent findings as to their functioning and impact. Similarly, legitimate questions have been voiced as to the negative impact of SPCs on timely access to affordable medicines.

On the basis of an analysis of three medicines for hepatitis C and cancer treatments, the present article critically engages with the policy justifications underlying SPCs. It then analyses access challenges to a hepatitis C medicine and an HIV treatment in Europe, highlighting the social cost of the introduction of SPCs. Both the normative and empirical analyses have demonstrated that the common justifications supporting the SPC regime are deeply questionable. The addition of SPC exclusivity has also heavily delayed competition and maintained high medicines prices in European countries. Ultimately, the granting of such extended exclusive private rights on medicines may result in unnecessary suffering and be a factor in the erosion of access to medicines for all.

## Background

In the context of increasing financial pressure on health care systems within European countries, some European countries recently introduced treatment rationing (eg France, Switzerland) [1, 2]. Together, these developments increasingly threaten the sustainability of healthcare systems [3]. In 2016, the Council of the European Union (EU) invited the European Commission to provide an analysis on the EU pharmaceuticals incentives package [4]. Included in the review was the EU Supplementary Protection Certificate (SPC), a mechanism created in 1992 to provide additional marketing exclusivity following a given medicine’s patent expiration.

A heated debate occurred throughout the review. The originator position firmly holds that extended market exclusivity is critical to securing growth and incentivising research and development (R&D) [5]. This position is grounded in the notion that pharmaceutical development is a high-cost, high-risk, stringently-regulated process effectively resulting in a much shorter exclusivity period in the market than the 20-year patent term. The SPC system is hence argued to provide additional guarantee and incentive [6–9]. By contrast, the European generic industry argued that the SPC regime hindered their global competitiveness [10]. Answering generic undertakings’ request for change, the Regulation was amended and Article 5 now allows generic production for export to third-countries (‘manufacturing waiver’) as a derogation and a ‘storing’ option permitting generic production 6 months pre-SPC expiration to prepare their products for market launch in the EU [11].

For their part, civil society organisations pointed to public health-related considerations, including the negative impact of SPC-extended market exclusivity on the affordability of medicines, whereby high prices are maintained which intensify concerns regarding the sustainability of medicines supply required for the treatment of all patients [12, 13]. While the five studies commissioned and published by the Commission in 2017 and 2018 throughout the SPC review investigated the origin, practices, economic rationale, impact, and legal aspects of SPCs [14–18], they did not analyse the social impact of SPCs in depth from the perspective of securing and protecting the sustainable provision of health care to all patients in need. In light of these issues, key questions addressed in the present review include whether and to what extent SPCs and other pharmaceutical incentive measures strike the correct balance of interests and the extent to which SPCs may hinder the availability and affordability of lifesaving medicines in Europe.

In order to engage in an evidence-based discussion of the social impact of SPCs, this article begins with a brief overview of the development of patents and other market exclusivity instruments and their impact on access to medicines. Section two introduces the specific case of SPCs, including the recent Commission review, while section three assesses SPCs as a means of offsetting R&D investments for selected medicines. The fourth section presents two recent cases of publicly-reported access to medicines challenges in a number of European countries. Finally, section five discusses the rationale and social cost of patent term extensions, such as SPCs, in light of the right to access medicines as an integral part of the realisation of the right to health for all.

## Methodology and scope

To critically assess the impact of SPCs on access to medicines, a review of the existing literature and several case studies are presented. For the literature review, resources were obtained from public libraries (eg the British Library and the Institute of Advanced Legal Studies Library), websites, and online databases (eg Westlaw, HeinOnline, JSTOR, and LexisNexis). The assumption underpinning the SPC regime is that SPCs are necessary to provide a ‘period of effective protection’ ‘sufficient to cover the investment put into the research’ [19]. To test this presumption, sofosbuvir, trastuzumab, and imatinib were selected as case studies. These drugs were chosen for three main reasons: they all have high therapeutic value, are included in the World Health Organization (WHO) Model List of Essential Medicines, and have been made available at expensive prices whilst generating enormous revenues as ‘blockbuster’ medicines. For the three medicines, patent and SPC status, sales revenue, and R&D investment were reviewed. Publicly available information was relied upon: sales revenue data is derived from originator supplier financial reports, and data on investments per product was found in literature, company reports and media outlets. To assess the social cost of high prices facilitated by market exclusivity, the cases of sofosbuvir and the fixed-dose combination tenofovir disoproxil fumarate and emtricitabine (TDF/FTC) are dissected, using a variety of public sources.

## Introduction: medicines pricing and SPCs

To undergo a discussion and analysis of the impact of SPCs on access to medicines in Europe, it is imperative to comprehend the evolution of international law on pharmaceutical patents, a processes characterised by constant pressure for longer periods of exclusivity protection by the pharmaceutical industry [20]. Before 1992, a number of European countries did not provide patent protection on pharmaceutical products [21]. At the international level, the World Trade Organization (WTO) Agreement on Trade-Related Aspects of Intellectual Property Rights (TRIPS) entered into force in 1995 [20, 22]. Critics have convincingly argued that TRIPS was drafted by lawyers and economists in the interests of US-based corporations [23], illustrating the setting of a ‘neo-liberal agenda of global governance’ [20, 23]. Among other provisions, TRIPS unified the patent terms in different countries, requiring 20 years’ patent protection from the date of filing (Article 33) [22]. Consequently, TRIPS extended patent terms under most national laws, which previously had often ranged from 15 to 17 years, although in some cases they could be as short as 5–7 years [24]. Despite the extended protection period, pharmaceutical sector lobbying persisted seeking even longer exclusivity by resorting to various legal and regulatory means, including patent term extensions or restorations.

At the domestic level, one of the most influential examples in the early shift towards the extension of pharmaceutical patent terms was the US Drug Price Competition and Patent Term Restoration Act 1984, often known as the Hatch-Waxman Act [25]. This Act provided an extension of the patent term of up to 5 years, thus enabling up to 14 years of an effective statutory monopoly as of the marketing date [26]. The Hatch-Waxman Act had far-reaching effects, particularly the legislative reasoning for granting this extended exclusivity – *ie* to compensate for marketing time lost fulfilling regulatory requirements and to recover R&D investments [26, 27]. In the 1990s in Europe, several countries adopted similar legislation that extended pharmaceutical market exclusivity status upon patent expiry in the form of SPCs, *eg* in France and Italy [28, 29]. To preserve the integrity and the functioning of the common market, the European Economic Community established a uniform system for granting SPCs in 1992 [19]. SPCs are not, however, the only EU mechanisms offering protection beyond patent expiry; other types not dealt with in this study include the paediatric extension, orphan market exclusivity, data exclusivity, and market protection. Such additional protections go beyond the obligations imposed by TRIPS and are hence typically referred to as ‘TRIPS-plus’ provisions. The EU systematically tries to impose SPC-type provisions in other countries via bilateral trade negotiations.

Many such TRIPS-plus provisions have proven detrimental to accessing affordable medicines in Europe [30] and elsewhere [31]. For instance, a study on the public health impact of introducing patent term extensions in Thailand found that a five-year market exclusivity extension would result in an annual increase in medicines expenditure from USD 146.3 to 696.4 million [32]. Similarly, a study on existing Australian patent term extensions demonstrated that their elimination could save up to AU $241 million per year on public pharmaceutical expenditure [33]. Following the 2017 conclusion of the Canada-EU Comprehensive Economic and Trade Agreement (CETA) and the consequent adoption of an up to 2 years’ ‘certificate of supplementary protection’ (CSP) following patent expiry [34], a retrospective Canadian Parliament study estimated the CSP regime would have led to an increase in annual medicines expenditure of CA $392 million (€260 million) if it had been adopted 2 years earlier [35]. The latter examples concern high-income countries with largely similar market structures to those found in Europe, serving as appropriate comparisons for the purposes of our discussion and analysis of the actual cost of introducing market exclusivity mechanisms.

## SPCs in the European Union

An SPC is a special intellectual property (IP) right (title) available for medicinal products, including chemical pharmaceutical products and biological medicines that require authorisation by national regulatory authorities before they can be marketed. SPCs are granted by national patent offices (NPOs) based on the SPC Regulation [36]. The conditions for the grant of an SPC are outlined in the Regulation, requiring inter alia that the SPC be requested within 6 months of the medicine’s first market authorisation (Article 7(1)), which is protected by a ‘basic patent in force’ (Article 3(a)), or within 6 months from the grant of the patent if this occurred after the market authorisation was awarded (Article 7(2)). In addition, the product must not have already been the subject of an SPC (Article 3(c)). If these conditions are satisfied, the SPC shall take effect at the end of the 20-year term of the basic patent for a period equal to the period which elapsed between the filing date of the basic patent and the date of the first market authorisation of the medicine, reduced by a period of 5 years (Article 13).

Strictly defined, an SPC is not a patent term extension, as that would require amending EU Member State national patent laws and the European Patent Convention; nevertheless, the effects of SPC-derived exclusivities are identical to those derived from patents. In other words, SPCs de facto enable right-holders to maintain monopoly prices and effectively function identically to a patent extension or restoration. The rationale for introducing this extension scheme is to make up for exclusivity time lost between patent registration and marketing authorisation, since the former usually occurs several years before the latter [15]. Some argue that this time needs to be *restored* to ensure sufficient return on costly pharmaceutical R&D expenditures [37].

In 1992, the SPC Regulation, as codified in 2009, created a scheme for medicinal products ‘to provide adequate effective protection’ and reduce ‘the risk of research centres … relocating to countries that already offer greater protection’ [19]. To have ‘adequate effective protection’ under the Regulation, ‘the holder of both a patent and a certificate should be able to enjoy an overall maximum of fifteen years of exclusivity’ from the marketing authorisation [19]. The Regulation assumed that otherwise, ‘the period of effective protection under the patent [was] insufficient to cover the investment put into the research’ [19]. Between 2010 and 2016, 86% of new medicines introduced had an SPC in at least one country, and SPC protections have been filed on average in 18–19 Member States [18]. Meanwhile, concerns surrounding pharmaceutical expenditure and the corresponding threat to healthcare systems’ sustainability have grown, with medicines constituting 17.1% of the total health expenditure in the EU and 1.41% of GDP in 2014 [38]; moreover, new medicines spending outpaces growth in GDP and other healthcare expenditures [39]. In addition, the Commission acknowledges that ‘public and private payers increasingly grapple with how to afford the rising number of new and often expensive medicines’ [3]. In June 2016 the Council of the EU under the Dutch presidency expressed ‘concern about examples of a market failure...where patients access to effective and affordable essential medicines is endangered by very high and unsustainable price levels’ and concluded ‘that the incentives in this specific legislation need to be proportionate to the goal of encouraging innovation, improving patients’ access to innovative medicines with therapeutic added value and budgetary impact’ [4]. Accordingly, the Council requested the Commission undergo an evidence-based analysis of the impact of some current incentive mechanisms – including SPCs – on innovation, availability, and accessibility of medicinal products [4]; two of the studies procured by the Commission and published in May 2018 specifically investigated the economic impact and legal aspects of SPCs [14, 15].

Of the studies procured by the Commission, that by Copenhagen Economics (CE) provides an extensive overview of the functioning, use, and impact of the various IP incentives in Europe, including SPCs [15]. The study by the Max Planck Institute (MPI) examines the functioning of the SPC system from a legal perspective [14]. The latter study points to significant divergence between the CJEU’s interpretation of the SPC Regulation and NPO practice, and also between current practice and the original intentions and limitations in respect to SPCs. The study therefore calls for greater coherence in the granting of SPCs, a finding echoed by a Technopolis report requested by the Dutch government [40]. The Commission has advanced the idea of a unitary SPC title at the EU level, in place of national SPCs, in accordance with the new unitary patent system of the European Patent Office [41].

On a number of critical issues, however, the studies reached different conclusions. CE presents a positive co-relation with the average effective protection period in countries of export and domestic pharmaceutical R&D spending [15], while the MPI study reasoned that an increase in innovation after the introduction of SPCs *‘*does not imply a cause-effect relationship with the enactment or the amendment of that specific regulation’ [14]*.* The Technopolis study could not confirm the incentivising effect of SPCs since the factors driving pharmaceutical R&D expenditure could not be identified, also noting that the SPC regulation ‘does not contain any provisions to favour innovation originating from Europe over that from elsewhere’ [40]. The latter report also points to alternative incentives such as prizes and conditional investments in basic research as effective means to spur innovation.

Additionally, the MPI study questions whether the availability of patent or SPC protection affects companies’ decisions to locate research facilities in one jurisdiction or another, emphasising that other factors are likely of greater importance [14]. Only CE argues that SPCs could play a role in attracting innovation to Europe, while conceding that taxation, education, and other factors are probably more significant in that respect [15].

The Technopolis case studies into atorvastatin, omeprazole, and losartan estimated the total cost of SPCs to the Dutch healthcare system to be between €120–660 million for each medicine [40]. The MPI and CE studies both note that the SPC-induced generic entry delay may negatively impact healthcare budgets [14, 15]. The CE study estimated that shifting 10% of total spending from originator to corresponding generic products would generate a saving of USD 12.4 billion (€11 billion), *ie* 1% of the EU healthcare spending [14].

Combined, the three studies demonstrate the degree of inconclusiveness surrounding the SPC regime’s impact as an innovation incentive and limited (or non-existent) influence in attracting R&D activities to Europe. Absent from the existing reviews of the EU SPC system, however, are two elements: (1) whether companies objectively need SPCs to recover R&D investments and (2) an assessment of the social cost of the SPC Regulation, further developed below.

## SPCs: a fair mechanism to recover R&D investments?

This section critically assesses the grounds and regulatory objectives advanced to justify granting additional exclusivity rights, whether SPCs are necessary to recover R&D costs, and whether without SPCs the much-needed R&D on medicinal products would not be undertaken within the EU.

The claim that R&D investments cannot be recovered without extended exclusivity is frequently relied upon when SPCs or patent term extensions more generally are discussed, but supporting evidence remains scant. The key question is whether the patent holder cannot generate sufficient sales revenue to match or surpass the R&D investment from market approval to patent expiry, and hence whether an exclusivity extension to prevent generic competition should be implemented to do so. This hence calls for R&D investment calculations and investigating whether the 20-year patent term is indeed too short to serve its intended purpose.

Defining the R&D investment for a particular medicine is hampered by a lack transparency in pharmaceutical markets, particularly as to cost and R&D investment information for individual products. There is an ongoing debate related to R&D costs [42, 43], with estimates for the cost of bringing a new product to the market ranging from USD 320 million to 2.7 billion (adjusted for 2017 USD) [44]. An important factor in this context is what exactly constitutes an individual product’s R&D ‘cost’, which could arguably be limited to expenses directly linked to developing the individual medicine. Yet, industry and certain scholars argue that the cost of failures and opportunity costs should also be included, leading to much higher figures [42, 45]. A 2017 analysis of ten companies and drugs by Prasad and Mailankody [44], however, found the median cost of developing a new cancer medicine was USD 648 million, including failures; when opportunity costs were added, the median cost was USD 793.6 million [46]. The authors also found that 90% of the medicines’ sales revenues had surpassed R&D spending a median of 4 years after market approval (ranging from 0.8–8.8 years), and 80% when failure and opportunity costs were included [44].

In the absence of exact R&D expenditure data from pharmaceutical companies on the three medicines selected for this study, publicly available information is used to establish proxy indicators for the amount of investment required to bring a product to market. It was not possible to verify whether the cited figures include failure or opportunity costs.

For Solvadi®, Herceptin®, Gleevec/Glivec® sales revenues are compared with companies’ claimed R&D investments and the length of time for which the product was marketed. Table 1 provides an overview of originator companies’ annual sales of three high-priced drugs over the past 14 years. It should also be noted that, while Gleevec® was first marketed in 2001, differing data exists for the first 3 years’ sales revenue; for accuracy, this study therefore only focuses on data from 2004 onward.

**Table 1:** Reported global annual product sales by calendar year for selected medicines according to company annual reports (2004–2017) (in million USD)^a^

Year	Sovaldi® (sofosbuvir) Gilead [47]	Herceptin® (trastuzumab) Roche & Genentech [48]	Gleevec/Glivec® (imatinib) Novartis [49]
2004	N/A	1162	1634
2005	N/A	1717	2170
2006	N/A	3142	2554
2007	N/A	4027	3050
2008	N/A	4736	3944
2009	N/A	4845	3944
2010	N/A	5212	4265
2011	N/A	5936	4659
2012	N/A	6301	4675
2013	139	6565	4693
2014	10,283	6840	1237
2015	5276	6800	1219
2016	4001	6918	3323
2017	964	7154	1943
Total	20,663	34,277	43,310

^a^Amounts rounded to millions. Trastuzumab sales have been converted from CHF to USD using historical exchange rates

### Sofosbuvir and Gilead

Sofosbuvir, a highly effective direct-acting antiviral (DAA) medicine for treating hepatitis C infections, was first marketed by Gilead as Sovaldi® and is one of the most expensive pharmaceutical products in the US [50]. Just 1 year after launch, in 2014 Gilead reported USD 10.3 billion in global sales of this product [47], and over USD 20 billion from 2014 to 2017, as indicated in Table 1. Gilead’s actual R&D investment in sofosbuvir is unknown, though it acquired sofosbuvir through a USD 11 billion acquisition of Pharmasset in 2011 when Phase III trials on sofosbuvir were nearing completion [51]. The medicine was approved by the US Food and Drug Administration (USFDA) in December 2013 [52], and by the European Medicines Agency (EMA) in January 2014 [53]. Within the first 3 years after market launch, Gilead’s sales revenue for sofosbuvir surpassed the cost of the Pharmasset acquisition, and in just 5 years the sales revenue was almost double this investment.

In Europe, Gilead was granted a patent on sofosbuvir prodrug by the European Patent Office (EPO) [54], due to expire in 2028, in addition to a patent on the base compound [55]. Some Member State NPOs also granted Gilead SPCs that expire in 2029 [56–59]. Whether Gilead in fact needs an additional 9 months exclusivity to recover its sofosbuvir-related investment is doubtful: within 5 years of market approval (and long before the primary patent expiration) sofosbuvir sales revenues are nearly double the company’s disclosed investments in the Pharmasset acquisition.

### Imatinib and Novartis

The second example concerns the R&D investment-SPC relationship for imatinib, sold by Novartis as Gleevec® or Glivec® (depending on the country). When the drug was first approved in 2001, the price was set around USD 30,000 for a years’ supply. With an expected annual sales revenue of USD 900 million for imatinib in the US alone, the company would have been able to retrieve imatinib R&D investments within the drug’s first 2 years on the market [60]. Novartis allegedly aimed for this price, since the potential life-prolonging benefit remained uncertain [61]. However, as the medicinal benefit of the drug became clear the company increased its price, which reached USD 92,000 per year in 2012 in the US [61]. This increase was publicly denounced by more than 100 experts, including scientists involved in imatinib’s discovery [61]. According to company reports (Table 1), imatinib generated over USD 43 billion globally from 2004 to 2017.

Novartis was granted a patent by the EPO on the basis compound of imatinib, which expired in 2013 [62]. In addition, Novartis secured an SPC which expired in 2016 (eg in the Netherlands [63] and France [64]). As the sales revenue from the first 2 years after the launch of imatinib (ie 2001 and 2002), not included in the table above, were expected to suffice to recover the R&D investment, it is unlikely Novartis actually needed three additional years of market exclusivity in order to cover its R&D investment.

### Trastuzumab and Roche

The third example relates to trastuzumab, a drug sold by Roche as Herceptin® for the treatment of breast cancer developed by Genentech. In 1998 the drug successfully passed Phase III clinical trials and was undergoing fast-tracked USFDA approval when Genentech signed a licensing agreement in which Roche obtained all rights to license the drug outside the US [65]. The agreement stipulated that Roche was to pay a USD 40 million upfront fee with cash milestones for product development activities, that global development costs were shared, and that Genentech was to receive royalty payments [66]. In 1999 when trastuzumab entered the market, a sales revenue of CHF 300 million [67], roughly USD 200 million at the historical exchange rate [68] and equivalent to five times Roche’s upfront investment of USD 40 million was reported for the medicine. Between 2004 and 2017, trastuzumab sales accumulated to USD 34 billion (Table 1).

In Europe, Roche secured patent protection for trastuzumab, which expired in 2012 [69]; several countries granted SPCs extending exclusivity a further 2 years [70–72]. The full details of Roche’s investment to acquire the trastuzumab licence are undisclosed, though it appears to have been part of an extended arrangement [67] which ultimately led to Genentech’s acquisition by Roche in 2009 [73]. While acknowledging these limitations, the idea that a two-year SPC was necessary to recover trastuzumab investment costs becomes questionable when sales exceeded five times the upfront investment to acquire the licence for trastuzumab in the first year it was marketed.

**Table 2:** Key dates and relevant protection periods for sofosbuvir, imatinib, and trastuzumab, based on SPCs granted in France [56, 64, 71]

Product	Sofosbuvir	Imatinib	Trastuzumab
Year of market launch	2013	2001	1999
Year sales revenue surpassed R&D investment	2015	2003	2000
Basic patent expiry date	26/03/2028	25/03/2013	15/06/2012
SPC term	> 9 months	> 3 years 8 months	> 2 years 1 month
SPC expiry date	17/01/2029	21/12/2016	29/07/2014

Based on the available data, the justification for SPCs that patent protection periods are insufficient to cover R&D investments [19] appears deeply flawed for all three medicines investigated. In each case, the sales revenues surpassed the companies’ R&D investments within 3 years after market launch and more than 10 years before the end of the basic patent term. The TRIPS-agreed 20-year patent term would have been more than sufficient to recover the relevant R&D investment, proving that the assumed general need for SPCs is incorrect for some (and potentially more) medicines. These findings also strongly challenge the relevance of temporal considerations in the SPC approval process. Costs are the only relevant factor in whether a company has been able to off-set its investment to develop or acquire a medicine, requiring precise details on the cost structure of a medicine’s development and a company’s own investments.

## High drug prices eroding access to medicines

Ensuring access to all medicines by patients in need is a core human right obligation of the states in light of the right of health [74]. Multiple factors impede access to medicines, price being one of crucial prominence, as highlighted by a recent UN report [75]. Whereas prices that are ‘too low’ have resulted in the limitation or cessation of product supply, prices that are ‘too high’ have impeded the ability of healthcare systems to ensure medicines availability for all patients [76]. In May 2017, the WHO-Dutch government Fair Pricing Forum indicated that drug pricing has become a global issue, even affecting the wealthiest of countries [77]. While many factors hamper access to medicines in Europe (*eg* manufacturing quality issues or regulatory delays) the case studies below highlight how product price (enabled by patent or SPC market exclusivity) serves as a barrier to treatment and how medicines prices have threatened healthcare systems’ sustainability. While the first case study, sofosbuvir, is a fairly newer medicine, the second, TDF/FTC, is nearing the end of its market exclusivity meaning SPC considerations are especially relevant.

### Sofosbuvir

The highly-effective hepatitis C treatment sofosbuvir, discussed above, is marketed at high prices that severely financially burden health systems globally, including in European countries [78]. This results in patients being denied treatment access. In France, the total cost of sofosbuvir-based treatments was estimated to be 20% of total medicines expenditure in 2014 [79], which ‘put at risk on the medium term the sustainability of the health care system’ [80]. The government subsequently restricted treatment provision in 2015 to adults with the most severe conditions [79]. In 2016 these restrictions were formally lifted [81]. With an estimated 130,000 hepatitis C patients and treatment costing €28,000 per patient or more [82, 83], the cost of DAAs continues to debilitate the French health system. In Romania, approximately five hundred thousand to one million people are infected with hepatitis C, the highest burden in Europe, yet treatment was limited to 5800 patients from 2015 to 2016 [84]. In Italy, which also faces a heavy hepatitis C burden, in 2017 the Ministry of Health agreed to allow the personal use of unregistered generic medicines because the healthcare system could not afford treatment for all patients [85]. In Ireland, the 2017 budget of €30 million for the country’s hepatitis C programme was effectively spent halfway through the year, forcing authorities to deny newly-registered patients treatment [86]. Meanwhile, generic versions of key *DAAs*, including sofosbuvir, have become available on the global market, leading to generic competition and much lower prices [87]; the patent as granted is also under post-grant oppositions launched by civil society organisations and other actors [88]. Médecins Sans Frontières (MSF) announced at the end of 2018 that it has obtained generic hepatitis C treatments at a cost of €75 for a 12-week course [89].

Unless TRIPS flexibilities as voluntary or non-voluntary licences are considered and implemented, Gilead’s monopoly remains in effect in Europe until 2029, enabling the company to charge high prices beyond the expiry of the original patent in 2028. While the sofosbuvir SPC does not cause the documented access challenges it certainly appears contentious to grant such a certificate that will further prevent generic competition, given that sofosbuvir sales revenue surpassed Gilead’s R&D investment approximately 2 years after market entry.

### TDF/FTC

TDF/FTC, first marketed by Gilead as Truvada®, has long been used as an effective anti-retroviral medicine for treating HIV/AIDS; it has been recommended by the WHO to be used as a PrEP to protect at-risk individuals from contracting HIV [90]. TDF/FTC is a combination of tenofovir [91] and emtricitabine, both of which were developed by Gilead [92]. Gilead applied for SPCs for TDF/FTC in many European countries, which were due to expire in 2020 [93].

However, Gilead’s SPC was revoked in France and Germany [94, 95]. The SPC was also rejected in the Netherlands [96], as was a preliminary injunction request by Gilead to prevent generic competition in Ireland [97, 98]. In other countries, such as Denmark [99] and Switzerland, [100, 101] the SPC has been maintained. In Belgium, a court ruled in favour of Gilead [102]. These divergences in application of the SPC Regulation stemmed from uncertainties related to the definition of the condition of having a ‘basic patent in force’ to obtain an SPC, a question finally referred to the Court of Justice by the UK High Court in *Teva and others v Gilead* [103, 104].

In 2016, a European Centre for Disease Prevention and Control (ECDC) survey revealed that 31/32 European countries identified the cost of drugs as an issue preventing or limiting PrEP availability, and 24 rated the issue of high importance [105]. With no generic version available on the market at that time, the price of Truvada® was thus considered the main access barrier to the prophylaxis. As of October 2017, several generic versions of TDF/FTC had been EMA-approved [106] though they were not marketed in all Member States due to the effects of the associated SPCs. On 25 July 2018, the CJEU referred the case back to national courts signalling that the SPC should be revoked. Table 2 below shows the fragmentary availability of generic forms of TDF/FTC on the European market just prior to the ECJ decision.

The SPC status for TDF/FTC in different countries is indicated in Table 3, which also lists the prices of Truvada® and the cheapest generic forms according to the available information. As standardised pricing information is not available across Europe, the available pricing information differs greatly between countries. Pricing information in Table 3 should thus only be used to demonstrate the price differences between generic and originator versions within the same country. The table also details the availability of generics and whether TDF/FTC for PrEP was reimbursed in a country as of 23 July 2018.

**Table 3:** SPCs status of TDF/FTC and access to PrEP

TDF/FTC	The Netherlands	Belgium	UK	Denmark	Norway	France	Germany	Switzerland	Ireland
SPC	Rejected	Preliminary injunction rejected	Preliminary injunction rejected	Maintained	No information	Revoked	Revoked	Maintained	Preliminary injunction granted
Prices listed	Average retail price [107]	Ex-factory price (excluding taxes) [108]	Reference price [109]	Retail price [110]	Maximum retail price [111]	Ex-factory price (excluding taxes) [112]	Retail price [113, 114]	Reference price [115]	Retail price [116]
Truvada price	344	380	402	1110	550	295	819	773	400
Cheapest available generic price	48	N/A	N/A	N/A	243	139	53	N/A	85
Originator - generic price difference	86%	N/A	N/A	N/A	56%	53%	94%	N/A	79%
Generic available on the market	Yes [117]	No [103]	No [118]	No [99]	Yes [111]	Yes [119]	Yes [120]	No [115]	Yes [121]
Reimbursement	Yes [122]	Yes [123]	No [124, 125]	No information	Yes [126]	Yes [119]	Pending [127]	No [128]	No [129]

Prices are in euro for 30 oral doses of TDF/FTC on 23 July 2018. Local currencies were converted to euro on 23 July 2018 [130]

Where the SPC was rejected or revoked, affordable generic forms of TDF/FTC have become available, *eg* in Ireland, France and the Netherlands. Other than Belgium, none of the countries where the SPC was maintained reimburse PrEP. While no comprehensive data is available on the use of PrEP in the listed countries in function of the price, it can be reasonably assumed that few people can afford out-of-pocket expenses of €400 or more for monthly treatment. These high prices, combined with the lack of reimbursement, have compelled individuals to resort to internet purchases for generic alternatives [131]. In England, a generic version of the drug for PrEP use is only available as part of a clinical trial which has enrolled 10,000 people [125, 126]. However, as demand surpassed availability, the NHS started to facilitate importation, and sales of generics commenced in a London clinic earlier this year [132]. It is worth noting that generic TDF/FTC has been available on the global market for more than 10 years.

Overall, this analysis demonstrates that the *Truvada* decision can enable widespread access to generic versions of TDF/FTC in Europe [106]. With generic prices 53–94% lower in countries where available, the case may have a profound impact on the affordability of a PrEP programme, the reimbursement status of TDF/FTC as PrEP, and on the ability of users to pay for their own medicine.

## The social cost of SPCs

SPCs can also come at a ‘social cost’. While the concept of social costs is still matter of debate, it was described by K William Kapp as the economic problem of ‘tangible and intangible damages and losses caused by economic activities … not accounted for in the cost accounts of those responsible for their production, but are shifted to and borne by third persons, the whole community or future generations’ [133]. Kapp later enlarged the notion to include all damages and harmful effects of private and public decision-making if they are the result of the pursuit of a private gain [133]. In recent years, the factoring of social cost gained relevance in the context of *eg* environmental issues [134], but is also mentioned in respect to obstruction to competition through information hoarding via trade secrets claims [135]. As illustrated in Section four above, high medicines prices constrain the optimal use of state resources and compel states to resort to treatment rationing, undermining the enjoyment of the patient’s right to health. Besides individuals being denied treatment, potentially causing unnecessary suffering or death, the social cost of SPCs could be expressed as an opportunity cost, *ie* the impeded ability of healthcare systems to invest in other medicines, commodities, healthcare services, or quality of care. While outside the scope of this study, the extended social cost of SPCs appears particularly relevant for further research and policy considerations.

While SPCs are intended as innovation incentives, they can hinder the availability and affordability of lifesaving medicines. It therefore appears that this tool does not reflect the balance of interests originally intended between public health objectives and the private industry incentivisation. The evidence in this study raises valid concerns as to whether the granting of additional exclusive rights on medicines is eroding access to medicines for all, favouring profits not connected to the financing of R&D over social costs.

## Recommendations and conclusions

Based on this research as to the sales revenue of and R&D investments in a limited number of medicines (sofosbuvir, trastuzumab, and imatinib), it appears that the EU SPC regime may be based on a false premise that companies need longer exclusivity periods to compensate for the ‘loss’ of a period of effective protection during the market approval process, to enable them to recover R&D investments. The higher medicines prices associated with the generic competition delays caused by SPCs in relation to the three medicines analysed appears to be an unnecessary cost for society; this cost can be expressed in financial, but also ‘social’ terms. The SPC system can ultimately cause unnecessary suffering and/or death, as healthcare systems or patients are unable to afford essential medicines for patients.

The evidence provided in this study suggests a more thorough assessment of the assumptions underpinning the SPC system is required. Acknowledging that the above case studies cover a limited number of medicines, a similar study including more medicines is needed. In view of the current debates on IP incentives in Europe, it is crucial that the Commission provides appropriate evidence on this issue. Moreover, the obstacles to obtaining pharmaceutical companies’ actual R&D cost data further demonstrates the need for greater transparency to enable public scrutiny of healthcare expenditure. This study raises questions as to the public benefit of granting SPCs on medicines altogether, or at minimum calls for fundamental reform to the system and the way SPCs are granted. The conflation of the notions of time and cost in the granting of SPCs appears to be particularly problematic.

## Data Availability

All data generated or analysed during this study are included in this published article.

## Abbreviations

CE: Copenhagen Economics
CETA: Comprehensive Economic and Trade Agreement
CHF: Swiss franc
CSP: Certificate of Supplementary Protection
DAA: Direct-acting antiviral
ECDC: European Centre for Disease Prevention and Control
ECJ: Court of Justice of European Union
EMA: European Medicines Agency
EPC: European Patent Convention
EPO: European Patent Office
EU: European Union
GDP: Gross Domestic Product
IP: Intellectual Property
MPI: Max Planck Institute
MSF: Médecins Sans Frontières
PrEP: Pre-Exposure Prophylactic
R&D: Research and Development
SPC: Supplementary Protection Certificate
TDF/FTC: Tenofovir/Emtricitabine
TRIPS: Agreement on Trade-related Intellectual Property Rights
UK: United Kingdom
UN: United Nations
US: United States
USFDA: United States Food and Drug Administration
WHO: World Health Organization
WTO: World Trade Organization
